# Associação de Nível de Renda e Doença Isquêmica do Coração: Papel Potencial da Caminhabilidade

**DOI:** 10.36660/abc.20220844

**Published:** 2023-11-23

**Authors:** Rodrigo Julio Cerci, Miguel Morita Fernandes-Silva, João Vicente Vitola, Juliano Julio Cerci, Carlos Cunha Pereira, Margaret Masukawa, Ana Paula Weller Gracia, Lara Luiza Silvello, Pedro Prado, Murilo Guedes, Adriano Akira Ferreira Hino, Cristina Pellegrino Baena

**Affiliations:** 1 Quanta Diagnóstico por Imagem - Cardiovascular CT Curitiba PR Brasil Quanta Diagnóstico por Imagem - Cardiovascular CT, Curitiba, PR – Brasil; 2 Pontifícia Universidade Católica do Paraná Curitiba PR Brasil Pontifícia Universidade Católica do Paraná, Curitiba, PR – Brasil

**Keywords:** Doença Isquêmica do Coração, Renda, Imagem de Perfusão do Miocárdio

## Abstract

**Fundamento:**

O nível socioeconômico tem sido associado à doença isquêmica do coração (DIC). Bairros de alta renda podem expor os indivíduos a um ambiente construído que promova caminhadas para atividades diárias (caminhabilidade). Faltam dados sobre a associação entre renda e DIC em países de renda média. Também é incerto se a caminhabilidade medeia essa associação.

**Objetivos:**

Investigar se a renda está associada à DIC em um país de renda média e se a caminhabilidade dos bairros medeia a associação entre renda e DIC.

**Métodos:**

O presente estudo transversal avaliou 44.589 pacientes encaminhados para imagem de perfusão miocárdica (SPECT-MPI). A renda e a caminhabilidade foram derivadas do setor censitário residencial dos participantes. A pontuação quantitativa da caminhabilidade combinou as seguintes 4 variáveis: conectividade viária, densidade residencial, densidade comercial e uso misto do solo. A DIC foi definida pela presença de perfusão miocárdica anormal durante um estudo SPECT-MPI. Utilizamos modelos ajustados com efeitos mistos para avaliar a associação entre nível de renda e DIC e realizamos uma análise de mediação para medir o percentual da associação entre renda e DIC mediada pela caminhabilidade. Consideramos valores de p abaixo de 0,01 como estatisticamente significativos.

**Resultados:**

Dos 26.415 participantes, aqueles que residiam no setor censitário do tercil de menor renda eram mais fisicamente inativos (79,1% versus 75,8% versus 72,7%) quando comparados aos setores censitários do tercil de maior renda (p < 0,001). A renda foi associada à DIC ( *odds ratio* : 0,91 [intervalo de confiança de 95%: 0,87 a 0,96] para cada aumento de 1000,00 dólares internacionais na renda), para homens e mulheres igualmente (p para interação = 0,47). Os setores censitários com maior renda estiveram associados a uma melhor caminhabilidade (p < 0,001); no entanto, a caminhabilidade não mediou a associação entre renda e DIC (porcentagem mediada = −0,3%).

**Conclusões:**

A renda foi independentemente associada a maior prevalência de DIC em um país de renda média, independentemente de gênero. Embora a caminhabilidade tenha sido associada à renda do setor censitário, ela não mediou a associação entre renda e DIC.

**Visual Abstract:**
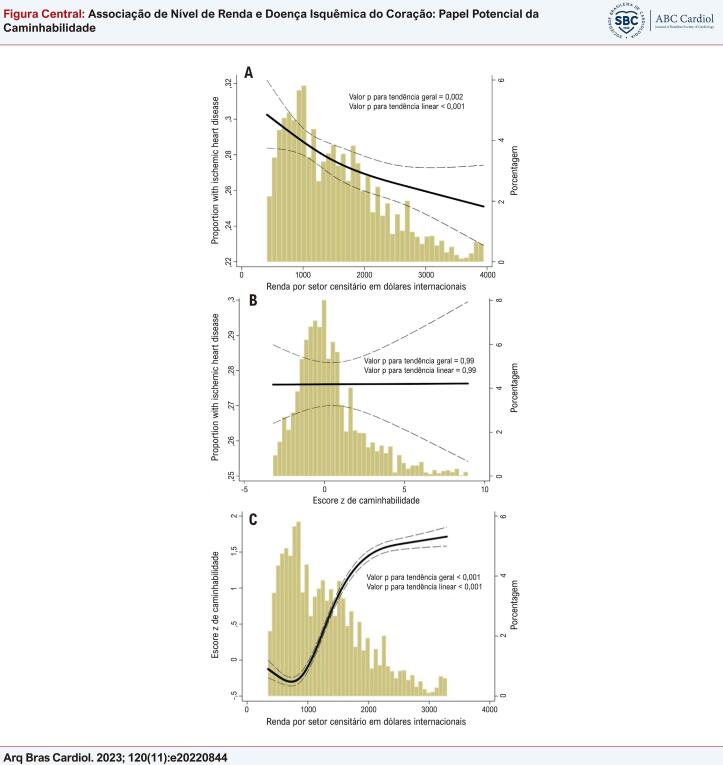
: Associação de Nível de Renda e Doença Isquêmica do Coração: Papel Potencial da Caminhabilidade

*Associação entre doença isquêmica do coração (ajustada por fatores de risco tradicionais e variáveis socioeconômicas) e nível de renda (A), e escore z de caminhabilidade (B), e associação de escore z de caminhabilidade e nível de renda (C).*

## Introdução

A doença isquêmica do coração (DIC) é responsável por 7,4 milhões de óbitos por ano ao redor do mundo, com um custo estimado de 2,1 bilhões de dólares apenas para tratamento agudo de complicações no Brasil.^1 , 2^ O diagnóstico de DIC está bem estabelecido, validado e disponível, usando imagem de perfusão miocárdica com tomografia computadorizada por emissão de fóton único (SPECT-MPI).^3 - 5^ Vários estudos determinaram o valor diagnóstico e prognóstico da perfusão miocárdica e da fração de ejeção do ventrículo esquerdo avaliadas por SPECT, para prever desfechos cardiovasculares adversos em vários subgrupos.^6 , 7^

O nível socioeconômico tem sido associado ao desenvolvimento de doenças cardiovasculares.^8^ Vários estudos demonstraram que o nível socioeconômico indiretamente influencia a DIC, impactando fatores de risco cardiovasculares comportamentais e metabólicos, fatores psicossociais e as condições ambientais de vida.^9 - 11^ A teoria da causação social e a teoria do conflito sugerem que problemas de saúde e comportamentais surgem quando recursos e recompensas são oferecidos de forma diferente para populações diferentes causando níveis diferentes de estresse. Tem sido verificada uma associação entre renda e DIC em países de alta renda, mas variáveis socioeconômicas como nível educacional, emprego, acesso à saúde e fatores psicossociais são frequentemente testadas em combinação, onde ainda faltam análise de mediação e dados sobre a causalidade direta de cada um destes fatores.^9 , 12^ Por exemplo, a desigualdade de renda tem sido associada ao aumento da criminalidade, o que também tem sido associado à redução da coesão social. A falta de segurança resultante da elevada criminalidade e da baixa coesão pode reduzir a atividade física ao ar livre, levando ao aumento da pressão arterial, do índice de massa corporal e de outros fatores de risco cardiovascular.^13^ Por outro lado, a atividade física regular está associada a melhor perfil de risco cardiometabólico e menor risco de eventos cardiovasculares maiores.^14 , 15^ Um ambiente construído que promova caminhadas para atividades diárias, também conhecido como melhor caminhabilidade, tem sido positivamente associado à atividade física geral.^16^ Alguns estudos demonstraram que pessoas que vivem em bairros com menor caminhabilidade têm taxas mais altas de fatores de risco cardiometabólicos, como diabetes, obesidade, hipertensão e estilo de vida sedentário, bem como um maior risco previsto de doença cardiovascular em 10 anos.^17 - 22^ Todos estes são fatores de risco bem conhecidos para DIC, mas ainda é incerto se a caminhabilidade pode mediar uma associação entre renda e DIC.

Finalmente, os dados da associação entre renda e DIC são inconsistentes nos países de renda baixa e média, que suportam a carga mais elevada de doenças cardiovasculares e têm estruturas sociais, ambientais e urbanas mais diversificadas quando comparados com países de renda alta.^23 - 25^ A magnitude diversa e a interpolação dessas variáveis socioeconómicas mensuráveis em países de renda mais baixa podem resultar em diferentes correlações com a DIC, e essas informações podem mudar expressivamente a alocação de recursos já escassos pelos formuladores de políticas públicas, que precisam se concentrar em políticas de alto impacto para reduzir a prevalência e a mortalidade da DIC.

Os objetivos do presente estudo foram: (1) investigar se o nível de renda está associado à DIC em um grande centro urbano de um país de renda média (PRM) e (2) testar se a caminhabilidade do bairro medeia a associação entre renda e DIC.

## Métodos

### População

Realizamos um estudo transversal que avaliou pacientes submetidos ao primeiro estudo SPECT-MPI clinicamente encaminhado, de fevereiro de 2010 a agosto de 2017, em um centro de imagem cardiovascular de alto volume localizado em Curitiba, Paraná, Brasil. Cada paciente consecutivo submetido a SPECT-MPI foi elegível para análise, a menos que algum dos seguintes critérios de exclusão fosse atendido: participantes não residentes em Curitiba, idade inferior a 18 anos, falta de dados de renda ou resultados inconclusivos do estudo SPECT-MPI. Curitiba é um grande centro urbano da Região Sul do Brasil, com 1.751.907 habitantes, onde 14,7% da população recebia menos que um salário mínimo de acordo com o censo populacional brasileiro mais atualizado (2010).^26^ De acordo com o mesmo censo, o Índice de Desenvolvimento Humano municipal foi de 0,823 e o índice de Gini, que representa a desigualdade de renda e varia de 0% a 100%, foi de 56%.^26^ O estudo foi aprovado pelo Comitê de Ética da Pontifícia Universidade Católica do Paraná (CAAE: 71331517.4.0000.00020) seguindo as normas internacionais e locais. Todos os dados individuais foram coletados e incluídos no registro da instituição durante o estudo SPECT-MPI, quando todos os indivíduos forneceram consentimento esclarecido para o uso dos seus dados para fins científicos.

### Coleta de dados para fatores tradicionais de risco cardiovascular

Uma enfermeira treinada entrevistou cada participante antes da aquisição das imagens. Foram coletados dados sobre idade, gênero, sintomas, histórico médico passado, fatores de risco cardiovascular e uso de medicamentos. Hipertensão, dislipidemia e diabetes mellitus foram definidos com base em diagnóstico prévio autorreferido ou uso de medicamentos anti-hipertensivos, hipolipemiantes ou antidiabéticos. Histórico familiar positivo de DIC prematura foi definido como parentes de primeiro grau com DIC de início precoce (homens ≤ 55 anos, mulheres ≤ 65 anos). A atividade física foi autorreferida e considerada como qualquer exercício aeróbico de pelo menos 30 minutos, 3 vezes por semana, para promoção da saúde, prevenção ou tratamento de doenças cardiovasculares. Os participantes foram considerados fisicamente inativos se não atenderam aos critérios acima. Também foram autorrelatados tabagismo, histórico prévio de DIC conhecida (infarto do miocárdio prévio, revascularização percutânea, cirurgia de revascularização do miocárdio ou DIC confirmada por angiografia coronária), altura e peso.

### Variáveis socioeconômicas e de caminhabilidade

As variáveis socioeconômicas foram coletadas do censo populacional brasileiro mais atualizado (2010) e os dados de homicídios foram obtidos da Secretaria de Segurança Pública do Estado do Paraná.^26^ O endereço de cada participante foi geocodificado usando uma plataforma on-line específica (Google Geocoding API Maps, Alphabet Inc, Estados Unidos), e variáveis individuais foram derivadas do setor censitário residencial do participante, incluindo: a renda média em moeda brasileira (real) por mês, nível de escolaridade definido pela alfabetização (analfabeto ou alfabetizado em qualquer nível) e nível de criminalidade estratificado pelo número de homicídios por 100 mil habitantes por ano. A renda foi posteriormente convertida para dólares internacionais (Int$), multiplicando-se a renda média em reais pela taxa unitária de paridade de poder de compra do Brasil do ano de 2010 (1,388).

A caminhabilidade foi medida para cada setor censitário por uma pontuação quantitativa que combina conectividade viária, densidade residencial, densidade comercial e uso misto do solo, obtidos por meio de camadas de dados, conforme descrito anteriormente.^16^ Os valores brutos de cada indicador foram então normalizados usando escores z. Finalmente, um escore z de caminhabilidade foi obtido pela média de cada indicador de escore z e usado como uma variável contínua, conforme descrito em outra publicação.^27^

### Aquisição e análise de SPECT-MPI

A variável de desfecho foi a presença de DIC no nível do participante, definida por perfusão miocárdica anormal durante o estudo SPECT-MPI. Todos os participantes foram submetidos a aquisições de imagens de estresse e repouso após injeção intravenosa de ^99m^Tc-sestamibi de 20 a 25 mCi ajustado ao peso. As imagens começaram 30 a 60 minutos após a injeção em repouso e 15 a 30 minutos após a injeção no pico de estresse. Foi realizada aquisição com protocolo de imagem convencional usando janelas de energia padrão para ^99m^Tc em câmeras gama de cabeça dupla com um colimador multifuncional de baixa energia.^6^ Nenhuma correção de atenuação foi usada.

Foi realizada a interpretação visual semiquantitativa do SPECT-MPI por consenso de 2 observadores experientes e certificados pelo conselho, usando cortes de eixo curto e eixo longo vertical, divididos em 17 segmentos padrão para cada paciente usando software específico (QPS, Cedars-Sinal, Los Angeles, Califórnia, Estados Unidos).^28^ Cada segmento foi pontuado com base na captação do traçador da maneira seguinte: 0, normal; 1, levemente reduzida; 2, moderadamente reduzida; 3, severamente reduzida; e 4, captação ausente do traçador em imagens de repouso e estresse. Um escore de estresse somado (SSS) foi obtido pela soma dos escores dos 17 segmentos das imagens de estresse. Os estudos foram classificados como normais (SSS < 4) ou anormais (SSS ≥ 4).

### Análise estatística

Os participantes foram divididos de acordo com tercis de renda por setor censitário, apenas para comparações de variáveis entre grupos. As variáveis contínuas foram testadas quanto à normalidade usando estatísticas de assimetria e curtose e apresentadas como média com desvio padrão, se distribuídas normalmente, ou mediana com intervalo interquartil, se não distribuídas normalmente. As variáveis categóricas foram apresentadas como proporções. Utilizamos ANOVA unidirecional com os tercis de renda como variável ordinal. Para variáveis binárias, utilizamos o teste qui-quadrado estendido de Mantel Haenszel para tendências lineares entre os tercis. Elaboramos um gráfico acíclico direcionado e o utilizamos como representação visual de suposições causais (Material Suplementar) para selecionar as variáveis para os modelos. Também utilizamos ponderação de probabilidade inversa em todos os modelos, contabilizando a distância entre o endereço dos participantes e o centro de imagem cardiovascular. Para considerar a correlação entre indivíduos que vivem no mesmo setor censitário, construímos modelos multiníveis (2 níveis) a partir de modelos de efeitos mistos para ajustar possíveis variáveis de confusão para avaliar a associação entre nível de renda (como variável contínua) e DIC e realizamos uma análise de mediação para medir o percentual da associação entre renda e DIC mediado pela caminhabilidade. No primeiro nível, foram incluídas as variáveis individuais (idade, gênero e fatores de risco cardiovascular). No segundo nível, foram incluídas variáveis derivadas do setor censitário (renda, caminhabilidade e alfabetização). Para análise foi utilizado o software Stata versão 15 (Stata Corp, College Station, Texas, Estados Unidos), e consideramos valores de p abaixo de 0,01 como estatisticamente significativos.

## Resultados

### Características da população

**Figura 1:**
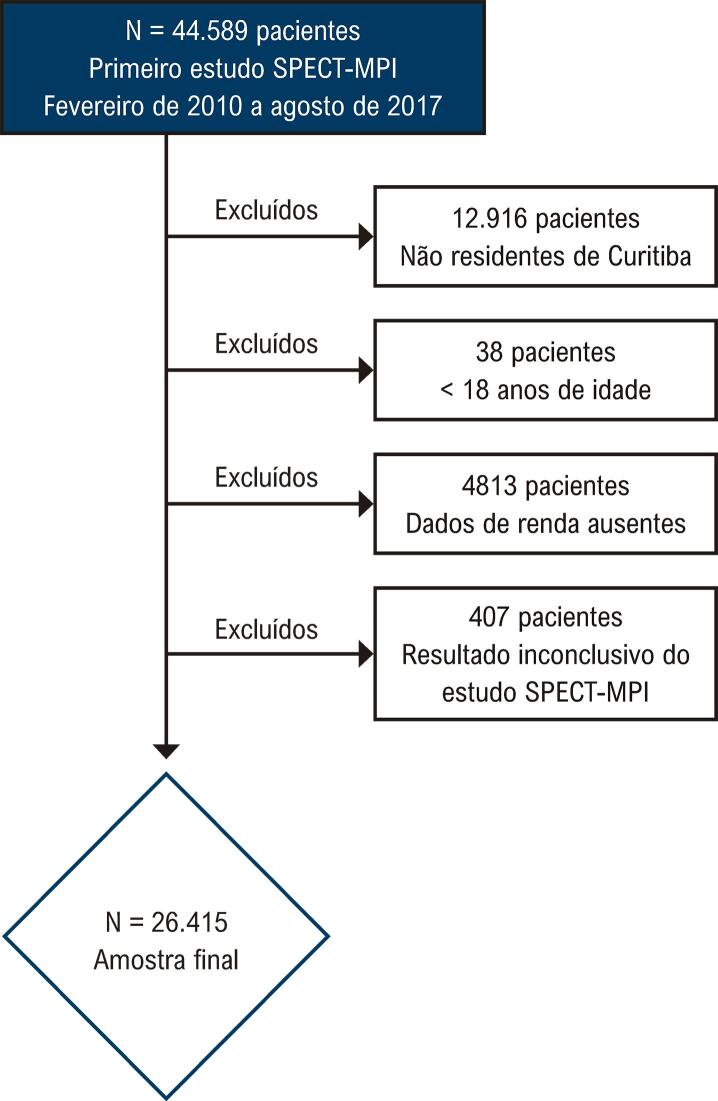
– Fluxograma dos critérios de exclusão

**Figura 2:**
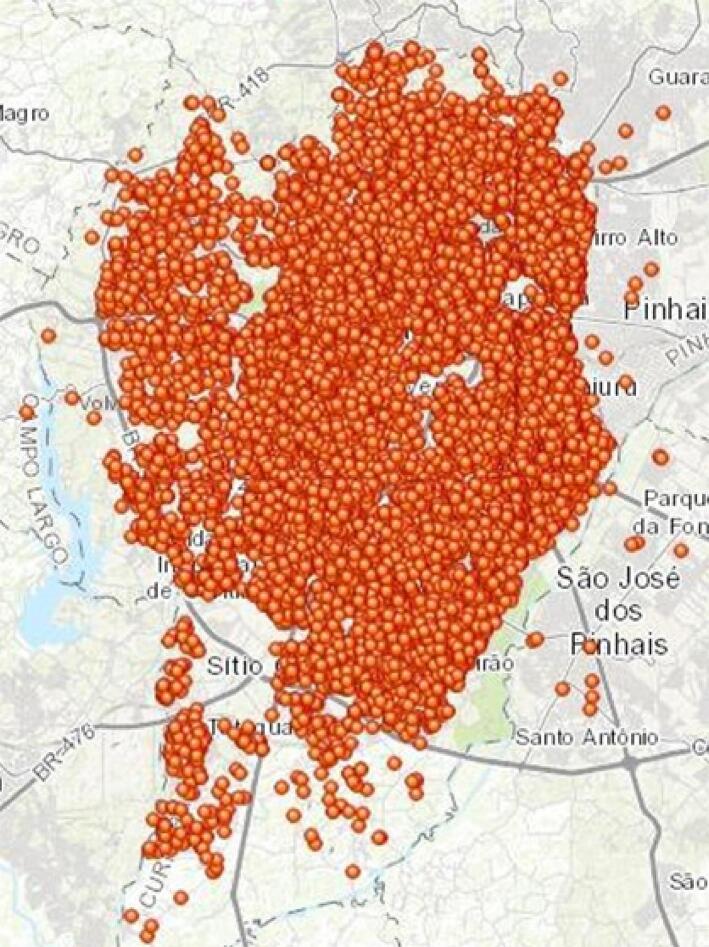
– Endereços geocodificados dos 26.415 participantes de Curitiba

Avaliamos 44.589 pacientes que foram submetidos ao primeiro estudo SPECT-MPI clinicamente encaminhado. Após aplicação dos critérios de exclusão, a amostra final foi composta por 26.415 pacientes que foram incluídos na análise ( Figura 1 ), sendo residentes em 2168 dos 2193 setores censitários de Curitiba ( Figura 2 ). A maioria dos endereços dos participantes ficava a 10 km do centro de imagem (46,4% dentro de 5 km, 39,0% entre 5 e 10 km, 12,6% entre 10 e 15 km e 2% acima de 15 km). Os participantes estavam cobertos por planos de saúde privados (n = 25.623; 96,5%) e públicos (n = 792; 3,5%). As características clínicas da população estratificada pelos tercis de renda são apresentadas na Tabela 1 .

**Tabela 1:** – Características populacionais e resultados do SPECT-MPI por renda

Renda mensal	Primeiro tercil (Int$) (230,5 - 1010,7)	Segundo tercil (Int$) (1010,7 - 1791,9)	Terceiro tercil (Int$) (1791,9 - 5743,3)	
**Variáveis socioeconômicas por setor censitário**	n=1299	n=531	n=338	Valor p
Escore z de caminhabilidade, média ± DP	–0,52 ± 1,51	0,64 ± 3,01	1,79 ± 2,75	< 0,001
Proporção média de alfabetização, média ± DP	0,96 ± 0,02	0,99 ± 0,01	0,99 ± 0,01	< 0,001
Taxa de homicídios por 100 mil habitantes, mediana (IIQ)	81,9 (0,0, 180,5)	0,0 (0,0, 89,8)	0,0 (0,0, 0,0)	< 0,001
**Fatores de risco tradicionais por paciente**	n=8806	n=8809	n=8800	
Idade, anos, média ± DP	60,82 ± 12,36	63,08 ± 12,63	64,74 ± 12,26	< 0,001
Gênero feminino, n (%)	4586 (52,1%)	4283 (48,6%)	4103 (46,6%)	< 0,001
IMC, médio kg/m^2^ ± DP	28,38 ± 5,22	27,69 ± 4,74	27,43 ± 4,51	< 0,001
Hipertensão, n (%)	5752 (65,3%)	5463 (62,0%)	5241 (59,6%)	< 0,001
Diabetes, n (%)	2362 (26,8%)	2070 (23,5%)	2003 (22,8%)	< 0,001
Dislipidemia, n (%)	4629 (52,6%)	4660 (52,9%)	4724 (53,7%)	0,130
Histórico familiar de DIC prematura, n (%)	1610 (18,3%)	1572 (17,8%)	1459 (16,6%)	0,003
Tabagismo, n (%)	894 (10,2%)	797 (9,0%)	754 (8,6%)	< 0,001
Inatividade física, n (%)	6962 (79,1%)	6675 (75,8%)	6393 (72,7%)	< 0,001
**Histórico anterior de DIC conhecida**				
Revascularização percutânea prévia, n (%)	1302 (14,8%)	1297 (14,7%)	1256 (14,3%)	0,340
CRM prévia, n (%)	600 (6,8%)	519 (5,9%)	529 (6,0%)	0,028
IM prévio, n (%)	1053 (12,0%)	900 (10,2%)	784 (8,9%)	< 0,001
**Sintomas**				
Dor torácica atípica, n (%)	2561 (29,2%)	1996 (22,8%)	1686 (19,3%)	< 0,001
Dor torácica típica, n (%)	658 (7,5%)	513 (5,9%)	395 (4,5%)	< 0,001
**SPECT-MPI**				
Perfusão miocárdica anormal, n (%)	2771 (31,5%)	2682 (30,4%)	2564 (29,1%)	< 0,001

*CRM: cirurgia de revascularização do miocárdio; DIC: doença isquêmica do coração; DP: desvio padrão; IIQ: intervalo interquartil; IM: infarto do miocárdio; IMC: índice de massa corporal; Int$: dólares internacionais; SPECT-MPI: imagem de perfusão miocárdica com tomografia computadorizada por emissão de fóton único.*

### Associação entre nível de renda e doença isquêmica do coração

Após ajuste para possíveis fatores de confusão, o nível de renda foi inversamente associado à DIC ( Figura Central A), com *odds ratio* (OR) de 0,91 (intervalo de confiança [IC] de 95%: 0,87 a 0,96) para cada aumento de Int$ 1.000,00 na renda. Por outro lado, a caminhabilidade não foi associada à DIC (OR 1,00; IC 95%: 0,99 a 1,02), conforme mostrado na Figura Central B. Outros fatores de risco tradicionais, como diabetes, tabagismo, histórico familiar de DIC prematura e inatividade física, também foram associados à DIC. Diabetes teve a associação mais forte, com OR de 1,57 (IC 95%: 1,44 a 1,72), conforme Tabela 2 . Embora a DIC tenha sido mais prevalente em homens do que em mulheres (31,2% versus 29,5%, p = 0,002), a associação com nível de renda foi semelhante em ambos os sexos (p para interação = 0,47).

**Tabela 2:** – Modelo multinível de efeitos mistos tendo DIC como desfecho

Variável	OR	IC de 95%	Valor p
Renda por Int$1000	0,91	0,87 - 0,96	< 0,001
Caminhabilidade, escore z	1,00	0,99 - 1,02	0,720
Idade, anos	1,04	1,03 - 1,04	< 0,001
Gênero feminino	1,02	0,94 - 1,10	0,680
IMC, kg/m^2^	1,02	1,01 - 1,03	< 0,001
Hipertensão	1,09	1,00 - 1,19	0,050
Diabetes	1,57	1,44 - 1,72	< 0,001
Dislipidemia	0,96	0,89 - 1,04	0,350
Histórico familiar de DIC prematura	1,01	0,91 - 1,12	0,840
Tabagismo	1,32	1,16 - 1,51	< 0,001
Inatividade física	1,41	1,28 - 1,55	< 0,001
IM prévio	3,58	3,09 - 4,14	< 0,001
Analfabetismo por setor censitário	4,97	0,81 - 30,40	0,080

*DIC: doença isquêmica do coração; IC: intervalo de confiança; IM: infarto do miocárdio; IMC: índice de massa corporal; Int$: dólares internacionais; OR: odds ratio.*

### Efeito mediador da caminhabilidade na associação entre nível de renda e doença isquêmica do coração

Os setores censitários com níveis de renda mais baixos foram associados a escores z de caminhabilidade mais baixos (−0,52 [IC 95%: −0,60 a 0,44] versus 0,64 [IC 95%: 0,38 a 0,89] versus 1,79 [IC 95%: 1,49 a 2,08]) do tercil de renda inferior para o superior, respectivamente (βeta: 0,115 ± 0,002, p < 0,001), conforme mostrado na Figura Central C, mas a caminhabilidade não mediou significativamente a associação entre nível de renda e DIC (porcentagem mediada = −0,3%). Também testamos a influência da criminalidade nessa mediação e descobrimos que a caminhabilidade mediou 0% (IC 95%: 0% a 28%) da associação entre renda e DIC em setores censitários sem homicídios por 100 mil habitantes; e mediou 3% (IC 95%: 0% a 18%) em setores censitários com pelo menos 1 homicídio por 100 mil habitantes.

## Discussão

As principais conclusões da presente investigação podem ser resumidas da maneira seguinte: (1) o nível de renda está independente e inversamente associado à DIC em um grande centro urbano de um país de renda média; e (2) embora bairros com níveis de renda mais baixos estivessem associados a escores mais baixos de caminhabilidade, a caminhabilidade não explicou a associação entre nível de renda e DIC.

### Associação entre nível de renda e doença isquêmica do coração

As chances de um estudo SPECT-MPI anormal diminuíram em 9% para cada aumento de Int$ 1.000,00 na renda do setor censitário dos participantes. Tem sido verificado uma associação entre renda e DIC em países de renda alta, mas os dados têm sido inconsistentes em países de renda média.^9 , 12 23 , 24^ Dados de países de renda média vizinhos, Bósnia-Herzegovina e Sérvia, mostraram resultados opostos.^29 , 30^ Janković et al.^29^ não encontraram nenhuma associação entre renda e saúde cardiovascular global em Bósnia-Herzegovina, enquanto Vuković et al.^30^ encontraram uma associação direta entre renda e fatores de risco cardiovascular tradicionais na Sérvia, onde os participantes mais ricos apresentavam o maior risco de hipertensão e dislipidemia (OR: 1,32 [IC 95%: 1,08 a 1,62] e OR 2,71 [IC 95%: 2,05 a 3,59], respectivamente). Uma revisão sistemática de 53 estudos concluiu que a mortalidade por DIC é mais elevada entre a população mais rica da Índia, um país de renda baixa-média.^31^ Os nossos dados corroboram o conhecimento de que a associação entre renda e DIC pode estar presente nos países de renda média, independentemente dos fatores de risco tradicionais.

A razão pela qual não podemos extrapolar associações de países de renda alta para países de renda baixa e média é a estrutura social, ambiental e urbana diversa entre países e regiões que vai além do nível de renda de forma isolada. Um exemplo dessa diversidade é a distribuição da obesidade na população de diferentes países.^32^ A obesidade é um fator de risco cardiovascular bem conhecido, que se tornou epidémico entre a população pobre em países de renda alta como os Estados Unidos da América, mas ainda é uma doença da população rica em países de renda baixa, onde apenas a população com renda mais elevada tem acesso à dieta ocidental propensa à obesidade.^32^

O Brasil tem passado por uma transição epidemiológica nos últimos 30 anos, com um declínio geral nas doenças transmissíveis e uma carga crescente das doenças não transmissíveis, onde a DIC se tornou a principal causa de morte.^33^ No entanto, mesmo dentro do Brasil, diferentes estados enfrentaram essa transição em tempos diferentes. Embora os estados de renda mais alta das Regiões Sul e Sudeste tenham iniciado a transição mais cedo, os estados de renda mais baixa das Regiões Norte e Nordeste ainda estão em movimento, enfrentando um aumento na mortalidade por DIC.^33 , 34^ Curitiba está localizada na Região Sul do Brasil onde a transição epidemiológica está mais avançada, o que pode explicar uma associação entre renda e DIC mais semelhante àquela encontrada em países desenvolvidos.

### Efeito mediador da caminhabilidade na associação entre nível de renda e doença isquêmica do coração

O aumento do estresse individual é a explicação mais amplamente descrita para a disparidade de saúde por nível socioeconômico. Os indivíduos com renda mais baixa sofrem mais stress, incluindo insegurança na habitação, salário, acesso aos alimentos e segurança, ao mesmo tempo que têm menos recursos para lidar com esses desafios, o que leva ao aumento de comportamentos de risco, como tabagismo, abuso de álcool e inatividade física. Tal comportamento se traduz em maior prevalência de fatores de risco tradicionais e doenças cardiovasculares.^11^

Vários estudos também verificaram uma associação entre caminhabilidade e fatores de risco cardiovasculares, notadamente em países desenvolvidos e de alta renda, onde a inatividade física foi apontada como principal mediadora dessa associação.^17 - 20 , 35 - 38^ Visto que a população que reside em setores censitários de Curitiba com baixa caminhabilidade é menos fisicamente ativa,^16 , 21^ e visto que demonstramos no presente estudo que setores censitários com baixa caminhabilidade estão associados a uma população de baixa renda na mesma cidade, encontramos equilíbrio para testar se a caminhabilidade poderia mediar parte da associação entre renda e DIC. Até onde sabemos, este é o primeiro estudo a testar a possível mediação da caminhabilidade na associação entre nível de renda e DIC, que geralmente representa um desfecho patológico avançado da exposição de um paciente a uma combinação de muitos desses fatores de risco tradicionais, socioeconômicos e ambientais durante um longo período.^39^ Não encontramos uma mediação significativa da caminhabilidade na associação entre nível de renda e DIC.

### Limitações

As limitações principais do presente estudo estão relacionadas ao desenho transversal e ao viés inerente a tal análise, que pode ser mitigado pelo grande tamanho da amostra e pelo uso de um termo de ponderação de probabilidade inversa para considerar a distância entre

o endereço dos participantes e o centro de imagem cardiovascular (viés de seleção). Calculamos a variável de exposição de renda com base no endereço dos participantes no momento do estudo SPECT-MPI, não considerando há quanto tempo estavam expostos a essa renda. Por fim, pode haver algum viés de encaminhamento, uma vez que todos os pacientes foram clinicamente encaminhados para o SPECT-MPI e não foram amostrados aleatoriamente em cada setor censitário da cidade de Curitiba.

## Conclusões

Neste grande registro, em um grande centro urbano de um país de renda média, residir em um setor censitário de baixa renda foi independentemente associado a maior prevalência de DIC, independentemente de gênero. Embora a caminhabilidade estivesse diretamente associada à renda do setor censitário, ela não mediou a associação entre nível de renda e DIC.
