# Supplementary material for: Associação de Nível de Renda e Doença Isquêmica do Coração: Papel Potencial da Caminhabilidade
Source: Arq Bras Cardiol. 2023 Nov 23;120(11):e20220844. [Article in Portuguese] doi: 10.36660/abc.20220844 (PMC10697676; doi:10.36660/abc.20220844)
Supplement: Translation

# Translation: Association of Income Level and Ischemic Heart Disease: Potential Role of Walkability

**Authors:** Rodrigo Julio Cerci, Miguel Morita Fernandes-Silva, João Vicente Vitola, Juliano Julio Cerci, Carlos Cunha Pereira, Margaret Masukawa, Ana Paula Weller Gracia, Lara Luiza Silvello, Pedro Prado, Murilo Guedes, Adriano Akira Ferreira Hino, Cristina Pellegrino Baena

**Affiliations:** 1 Brazil Quanta Diagnóstico por Imagem - Cardiovascular CT, Curitiba, PR – Brazil; 2 Brazil Pontifícia Universidade Católica do Paraná, Curitiba, PR – Brazil

**Keywords:** Myocardial Ischemia, Income, Myocardial Perfusion Imaging

## Abstract

**Background:**

Socioeconomic status has been linked to ischemic heart disease (IHD). High-income neighborhoods may expose individuals to a walking-promoting built environment for daily activities (walkability). Data from the association between income and IHD is lacking in middle-income countries. It is also uncertain whether walkability mediates this association.

**Objectives:**

To investigate whether income is associated with IHD in a middle-income country and whether neighborhood walkability mediates the income-IHD association.

**Methods:**

This cross-sectional study evaluated 44,589 patients referred for myocardial perfusion imaging (SPECT-MPI). Income and walkability were derived from participants’ residential census tract. Walkability quantitative score combined 4 variables: street connectivity, residential density, commercial density, and mixed land use. IHD was defined by abnormal myocardial perfusion during a SPECT-MPI study. We used adjusted mixed effects models to evaluate the association between income level and IHD, and we performed a mediation analysis to measure the percentage of the income-IHD association mediated by walkability. We considered p values below 0.01 as statistically significant.

**Results:**

From 26,415 participants, those living in the lowest-income tertile census tract were more physically inactive (79.1% versus 75.8% versus 72.7%) when compared to higher-income tertile census tracts (p < 0.001). Income was associated with IHD (odds ratio: 0.91 [95% confidence interval: 0.87 to 0.96] for each 1,000.00 international dollars increase in income) for both men and women equally (p for interaction = 0.47). Census tracts with a higher income were associated with better walkability (p < 0.001); however, walkability did not mediate the income-IHD association (percent mediated = −0.3%).

**Conclusions:**

Income was independently associated with higher prevalence of IHD in a middle-income country irrespective of gender. Although walkability was associated with census tract income, it did not mediate the income-IHD association.

**Central Illustration:** Association of Income Level and Ischemic Heart Disease: Potential Role of Walkability

*Association of ischemic heart disease (adjusted for traditional risk factors and socioeconomics variables) and income level (A), and walkability z-score (B), and association of walkability z-score and income level (C).*

## Introduction

Ischemic heart disease (IHD) accounts for 7.4 million deaths per year worldwide, with an estimated cost of 2.1 billion dollars solely for acute treatment of complications in Brazil.^1 , 2^The diagnosis of IHD is well-established, validated, and available, using single-photon emission computed tomography myocardial perfusion imaging (SPECT-MPI).^3 - 5^Several studies have determined the diagnostic and prognostic value of myocardial perfusion and left ventricular ejection fraction evaluated by SPECT, to predict adverse cardiovascular outcomes in several subgroups.^6 , 7^

Socioeconomic status has been associated with the development of cardiovascular disease.^8^Several studies have shown that socioeconomic status indirectly influences IHD by impacting behavioral and metabolic cardiovascular risk factors, psychosocial factors, and environmental living conditions.^9 - 11^Social causation theory and conflict theory suggest health and behavioral problems arise when resources and rewards are offered differently to different populations causing different levels of stress. Association between income and IHD has been found in high-income countries, but socioeconomic variables such as educational status, employment, health access, and psychosocial factors are often tested in combination, where mediation analysis and direct causality data for each of these factors are still lacking.^9 , 12^For instance, income inequality has been linked to higher criminality, which has also been tied to reduced social cohesion. The lack of safety resulting from high crime and low cohesion may reduce outdoor physical activity, leading to increased blood pressure, body mass index, and other cardiovascular risk factors.^13^On the other hand, regular physical activity is associated with a better cardiometabolic risk profile and a lower risk of major cardiovascular events.^14 , 15^A walking-promoting built environment for daily activities, also known as better walkability, has been positively associated with overall physical activity.^16^Some studies have shown that people living in neighborhoods with lower walkability have higher rates of cardiometabolic risk factors, such as diabetes, obesity, hypertension, and sedentary lifestyle, as well as a higher predicted 10-year cardiovascular disease risk.^17 - 22^These are all well-known risk factors for IHD, but it is still uncertain whether walkability can mediate an income-IHD association.

Finally, data from the income-IHD association is inconsistent in low- and middle-income countries (MIC), which carry the highest burden of cardiovascular disease and have more diverse social, environmental, and urban structures when compared to high-income countries.^23 - 25^The diverse magnitude and interpolation of these measurable socioeconomic variables in lower-income countries may result in different correlations with IHD, and this information can dramatically shift the allocation of already scarce resources by policymakers, who need to focus on high-impact policies to reduce IHD prevalence and mortality.

The objectives of this study were: (1) to investigate whether income level is associated with IHD in a large urban center of a MIC and (2) to test whether neighborhood walkability mediates the income-IHD association.

## Methods

### Population

We conducted a cross-sectional study that evaluated patients who underwent a first clinically referred SPECT-MPI, from February 2010 to August 2017, at a high-volume cardiovascular imaging center living in Curitiba, Paraná, Brazil. Every consecutive patient submitted to SPECT-MPI was eligible for the analysis unless any of the following exclusion criteria were met: participants not residing in Curitiba, less than 18 years of age, missing income data, or inconclusive SPECT-MPI results. Curitiba is a large urban center in the South Region of Brazil, with 1,751,907 inhabitants, where 14.7% of the population received less than the minimum wage according to the most updated Brazilian population census (2010).^26^According to the same census, the municipal Human Development Index was 0.823 and the Gini index, a ratio that represents income inequality and varies from 0% to 100%, was 56%.^26^The study was approved by the Pontifícia Universidade Católica do Paraná Ethics Committee (CAAE: 71331517.4.0000.00020) following international and local regulations. All individual data were collected and included in the institution registry during the SPECT-MPI, when all the individuals provided informed consent to use their data for scientific purposes.

### Data collection for traditional cardiovascular risk factors

A trained nurse interviewed each participant before imaging acquisition. Data regarding age, gender, symptoms, past medical history, cardiovascular risk factors, and use of medications were collected. Hypertension, dyslipidemia, and diabetes mellitus were defined based on self-reported previous diagnosis or use of anti-hypertensive, lipid-lowering, or antidiabetic medications. Positive family history of premature IHD was defined as first-degree relatives with early onset IHD (men ≤ 55 years, women ≤ 65 years). Physical activity was self-reported and considered as any at least 30 minutes of aerobic exercise 3 times per week, for health promotion, prevention, or treatment of cardiovascular disease. Participants were considered physically inactive if they did not meet the criteria above. Smoking status, prior history of known IHD (prior myocardial infarction, percutaneous revascularization, coronary bypass graft surgery, or IHD confirmed by coronary angiography), height, and weight were also self-reported.

### Socioeconomic and walkability variables

Socioeconomic variables were collected from the most updated Brazilian population census (2010), and homicide data were obtained from the Department of Public Safety of the State of Paraná.^26^Each participant’s address was geocoded using a specific online platform (Google Geocoding API Maps, Alphabet Inc, United States), and individual variables were derived from the participant’s residential census tract including the following: the average income in Brazilian currency (real) per month, education level defined by literacy (illiterate or literate in any level), and criminality level stratified by the number of homicides per 100,000 inhabitants per year. The income was further converted to international dollars (Int$), by multiplying the average income in Brazilian reais by the purchasing power parity unit rate for Brazil from the year 2010 (1.388).

Walkability was measured for each census tract by a quantitative score combining street connectivity, residential density, commercial density, and mixed land use, obtained through data layers, as previously described.^16^Raw values for each indicator were then normalized using z-scores. Finally, a walkability z-score was obtained by averaging each z-score indicator and used as a continuous variable, as described elsewhere.^27^

### SPECT-MPI acquisition and analysis

The outcome variable was the presence of IHD at the participant level, defined by abnormal myocardial perfusion during the SPECT-MPI study. All participants were submitted to stress and rest imaging acquisitions after intravenous injection of weight-adjusted 20 to 25 mCi of^99m^Tc-sestamibi. Images started 30 to 60 minutes after injection at rest and 15 to 30 minutes after injection at peak stress. Conventional image protocol acquisition using standard energy windows for^99m^Tc in dual-head gamma cameras with a low-energy all-purpose collimator was performed.^6^No attenuation correction was used.

Semi-quantitative visual interpretation of SPECT-MPI was performed by consensus of 2 experienced, board-certified observers using short-axis and vertical long-axis slices, divided into 17 standard segments for each patient using specific software (QPS, Cedars-Sinal, Los Angeles, California, United States).^28^Each segment was scored based on the tracer uptake as follows: 0, normal; 1, mildly reduced; 2, moderately reduced; 3, severely reduced; and 4, absent tracer uptake in rest and stress images. A summed stress score (SSS) was obtained by adding the scores of the 17 segments of the stress images. Studies were classified as normal (SSS < 4) or abnormal (SSS ≥ 4).

### Statistical analysis

Participants were divided by income tertiles per census tract, only for variable comparisons between groups. Continuous variables were tested for normality using skewness and kurtosis statistics and presented as means with standard deviations if normally distributed or medians with interquartile ranges if not normally distributed. Categorical variables were presented as proportions. We used one-way ANOVA with the income tertiles as an ordinal variable. For binary variables, we used the extended Mantel Haenszel chi-square test for linear tendencies between the tertiles. We drafted a directed acyclic graph and used it as a visual representation of causal assumptions (Supplemental Material) to select the variables for the models. We also used inverse probability weighting in all models, accounting for the distance between the participant address and the cardiovascular imaging center. To account for the correlation between individuals living in the same census tract, we built multilevel (2 levels) models from mixed-effects models to adjust for potential confounding variables to evaluate the association between income level (as a continuous variable) and IHD, and we performed a mediation analysis to measure the percentage of the income-IHD association mediated by walkability. In the first level, the individual variables were included (age, gender, and cardiovascular risk factors). In the second level, variables derived from the census tract were included (income, walkability, and literacy). The software Stata version 15 (Stata Corp, College Station, Texas, United States) was used for analysis, and we considered p values below 0.01 as statistically significant.

## Results

### Population characteristics

We evaluated 44,589 patients who underwent a first clinically referred SPECT-MPI. After applying the exclusion criteria, the final sample consisted of 26,415 patients who were included in the analysis ( Figure 1 ), living in 2168 out of the 2193 census tracts of Curitiba ( Figure 2 ). Most participants’ addresses were within 10 km from the imaging center (46.4% within 5 km, 39.0% between 5 and 10 km, 12.6% between 10 and 15 km, and 2% more than 15 km). Participants were covered by both private (n = 25,623; 96.5%) and public health insurance (n = 792; 3.5%). The clinical characteristics of the population stratified by the income tertiles are presented in Table 1 .

**Figure 1:** – *Exclusion criteria flowchart.*

**Figure 2:** – *Geocoded addresses of the 26,415 participants in Curitiba.*

**Table 1:** – Population characteristics and SPECT-MPI results by income

Monthly income	First tertile (Int$) (230.5 - 1010.7)	Second tertile (Int$)(1010.7 - 1791.9)	Third tertile (Int$) (1791.9 - 5743.3)	
**Socioeconomic variables by census tract**	n=1299	n=531	n=338	Valor p
Walkability z-score, mean ± SD	–0.52 ± 1.51	0.64 ± 3.01	1.79 ± 2.75	< 0.001
Mean proportion of literacy, mean ± SD	0.96 ± 0.02	0.99 ± 0.01	0.99 ± 0.01	< 0.001
Homicide rate per 100,000 inhabitants, median (IQR)	81.9 (0.0. 180.5)	0.0 (0.0. 89.8)	0.0 (0.0. 0.0)	< 0.001
**Traditional risk factors by patient**	n=8806	n=8809	n=8800	
Age, years, mean ± SD	60.82 ± 12.36	63.08 ± 12.63	64.74 ± 12.26	< 0.001
Female gender, n (%)	4586 (52.1%)	4283 (48.6%)	4103 (46.6%)	< 0.001
BMI, kg/m^2^, mean ± SD	28.38 ± 5.22	27.69 ± 4.74	27.43 ± 4.51	< 0.001
Hypertension, n (%)	5752 (65.3%)	5463 (62.0%)	5241 (59.6%)	< 0.001
Diabetes, n (%)	2362 (26.8%)	2070 (23.5%)	2003 (22.8%)	< 0.001
Dyslipidemia, n (%)	4629 (52.6%)	4660 (52.9%)	4724 (53.7%)	0.130
Family history of premature IHD, n (%)	1610 (18.3%)	1572 (17.8%)	1459 (16.6%)	0.003
Smoking, n (%)	894 (10.2%)	797 (9.0%)	754 (8.6%)	< 0.001
Physical inactivity, n (%)	6962 (79.1%)	6675 (75.8%)	6393 (72.7%)	< 0.001
**Prior history of known IHD**				
Prior percutaneous revascularization, n (%)	1302 (14.8%)	1297 (14.7%)	1256 (14.3%)	0.340
Prior CABG, n (%)	600 (6.8%)	519 (5.9%)	529 (6.0%)	0.028
Previous MI, n (%)	1053 (12.0%)	900 (10.2%)	784 (8.9%)	< 0.001
**Symptoms**				
Atypical chest pain, n (%)	2561 (29.2%)	1996 (22.8%)	1686 (19.3%)	< 0.001
Typical chest pain, n (%)	658 (7.5%)	513 (5.9%)	395 (4.5%)	< 0.001
**SPECT-MPI**				
Abnormal myocardial perfusion, n (%)	2771 (31.5%)	2682 (30.4%)	2564 (29.1%)	< 0.001

*BMI: body mass index; CABG: coronary artery bypass graft; IHD: ischemic heart disease; Int$: international dollars; IQR: interquartile range; MI: myocardial infarction: SD: standard deviation; SPECT-MPI: single-photon emission computed tomography myocardial perfusion imaging.*

### Association of income level with ischemic heart disease

After adjusting for potential confounders, income level was inversely associated with IHD ( Central Illustration A), with an odds ratio (OR) of 0.91 (95% confidence interval [CI]: 0.87 to 0.96) for each Int$ 1,000.00 increase in income. On the other hand, walkability was not associated with IHD (OR: 1.00; 95% CI: 0.99 to 1.02), as shown in Central Illustration B. Other traditional risk factors, such as diabetes, smoking, family history of premature IHD, and physical inactivity, were also associated with IHD. Diabetes had the strongest association, with an OR of 1.57 (95% CI: 1.44 to 1.72), as shown in Table 2 . Although IHD was more prevalent in men than women (31.2% versus 29.5%, p = 0.002), the association with income level was similar in both genders (p for interaction = 0.47).

**Table 2:** – Multilevel mixed effects model having IHD as outcome

Variable	OR	95% CI	p value
Income per Int$1000	0.91	0.87 - 0.96	< 0.001
Walkability, z-score	1.00	0.99 - 1.02	0.720
Age, years	1.04	1.03 - 1.04	< 0.001
Female gender	1.02	0.94 - 1.10	0.680
BMI, kg/m^2^	1.02	1.01 - 1.03	< 0.001
Hypertension	1.09	1.00 - 1.19	0.050
Diabetes	1.57	1.44 - 1.72	< 0.001
Dyslipidemia	0.96	0.89 - 1.04	0.350
Family history of premature IHD	1.01	0.91 - 1.12	0.840
Smoking	1.32	1.16 - 1.51	< 0.001
Physical inactivity	1.41	1.28 - 1.55	< 0.001
Previous MI	3.58	3.09 - 4.14	< 0.001
Illiteracy by census tract	4.97	0.81 - 30.40	0.080

*BMI: body mass index; CI: confidence interval; IHD: ischemic heart disease; Int$: international dollars; MI: myocardial infarction; OR: odds ratio.*

### Mediation effect of walkability on the association of income level and ischemic heart disease

Census tracts with lower income levels were associated with lower walkability z-scores (−0.52 [95% CI: −0.60 to 0.44] versus 0.64 [95% CI: 0.38 to 0.89] versus 1.79 [95% CI: 1.49 to 2.08]) from the lower to the higher income tertile respectively (βeta: 0.115 ± 0.002, p < 0.001), as shown in Central Illustration C, but walkability did not significantly mediate the association between income level and IHD (percent mediated = −0.3%). We also tested the influence of criminality on this mediation and found that walkability mediated 0% (95% CI: 0% to 28%) of the income-IHD association in census tracts with no homicides per 100,000 inhabitants; and mediated 3% (95% CI: 0% to 18%) in census tracts with at least 1 homicide per 100,000 inhabitants.

## Discussion

The key findings of this investigation can be summarized as follows: (1) income level is independently and inversely associated with IHD in a large urban center in a MIC; and (2) although neighborhoods with lower income levels were associated with lower walkability scores, walkability did not explain the association between income level and IHD.

### Association of income level and ischemic heart disease

The odds of an abnormal SPECT-MPI decreased by 9% for each Int$ 1,000.00 increase in participant census tract income. Income-IHD association has been found in high-income countries, but data have been inconsistent in MICs.^9 , 12 , 23 , 24^Data from neighboring middle-income countries Bosnia-Herzegovina and Serbia showed opposite results.^29 , 30^Jankovic et al.^29^did not find any association between income and global cardiovascular health in Bosnia-Herzegovina, while Vukovic et al.^30^found a direct association between income and traditional cardiovascular risk factors in Serbia, where the richest participants had the highest risk of hypertension and dyslipidemia (OR: 1.32 [95% CI: 1.08 to 1.62] and OR: 2.71 [95% CI: 2.05 to 3.59], respectively). A systematic review of 53 studies concluded that IHD mortality is higher among the richest population in India, a low-middle-income country.^31^Our data build on the knowledge that income and IHD association may be present in MIC, regardless of traditional risk factors.

The reason why we cannot extrapolate associations from high-income countries to low- and middle-income countries is the diverse social, environmental, and urban structure between countries and regions that goes beyond income level alone. One example of this diversity is the distribution of obesity within the population of different countries.^32^Obesity is a well-known cardiovascular risk factor, which became epidemic among the poor in high-income countries like the United States of America, but it is still a disease of the rich in low-income countries where only the higher-income population has access to the obesity-prone western diet.^32^

Brazil has been undergoing an epidemiological transition over the past 30 years, with an overall decline in communicable diseases and an increasing non-communicable disease burden, where IHD became the leading cause of death.^33^Nevertheless, even within Brazil, different states have faced different timing in this transition. While the higher-income states in the South and Southeast Regions started the transition earlier, the lower-income states of the North and Northeast Regions are still on the move and facing an increase in IHD mortality.^33 , 34^Curitiba is located in the South Region of Brazil, where the epidemiological transition is more advanced, which may explain an income-IHD association more similar to the one found in developed countries.

### Mediation effect of walkability on the association of income level and ischemic heart disease

Increased individual stress is the most widely described explanation for health disparity by socioeconomic status. Lower-income individuals have more stress, including insecurity in housing, income, food access, and safety, while also having fewer resources to deal with these challenges, which leads to increased risky behavior, such as smoking, alcohol abuse, and physical inactivity. Such behavior translates into a higher prevalence of traditional risk factors and cardiovascular disease.^11^

Several studies also found an association between walkability and cardiovascular risk factors, notably in developed and high-income countries, where physical inactivity was pointed out as the main mediator of this association.^17 - 20 , 35 - 38^Since the population living in low walkability census tracts in Curitiba is less physically active^16 , 21^and since we demonstrated in this study that low walkability census tracts are associated with a lower-income population in the same city, we found equipoise to test whether walkability could mediate some of the income-IHD association. To the best of our knowledge, this is the first study to test the possible mediation of walkability on the income level-IHD association, which usually represents an advanced pathological endpoint of a patient’s exposure to a combination of many of these traditional, socioeconomic, and environmental risk factors over a long period.^39^We did not find a significant mediation of walkability on the income level-IHD association.

### Limitations

The main limitations of this study are related to the cross-sectional design and the inherent bias of such analysis, which may be mitigated by our large sample size and by the use of an inverse probability weighting term to account for the distance between the participant’s address and the cardiovascular imaging center (selection bias). We calculated the exposition variable income based on the address of the participants at the time of the SPECT-MPI study, not considering how long they had been exposed to that income. Finally, there may be some referral bias, since all patients were clinically referred for the SPECT-MPI and not randomly sampled from each census tract of the city of Curitiba.

## Conclusions

In this large registry, in a large urban center of a MIC, living in a low-income census tract was independently associated with a higher prevalence of IHD, irrespective of gender. Although walkability was directly associated with census tract income, it did not mediate the association between income level and IHD.
